# A Comparison of the Antibacterial Effects of Moringa Oleifera and Eucalyptus As Intracanal Irrigants on Enterococcus Faecalis: An in Vitro Study: Short title: Antimicrobial Efficacy of Herbal Products against E.faecalis

**DOI:** 10.31661/gmj.v15i.4141

**Published:** 2026-02-21

**Authors:** Sahar Shakoui, Shirin Fattahi, Ladan Paya, Parya Emamverdizadeh, Negin Neshanifard, Mohammad Ali Irani, Shaghayegh Ghadimi

**Affiliations:** ^1^ Department of Endodontics, Faculty of Dentistry, Tabriz University of Medical Sciences, Tabriz, Iran; ^2^ Department of Oral and Maxillofacial Pathology, Faculty of Dentistry, Tabriz University of Medical Sciences, Tabriz, Iran; ^3^ Department of Oral Medicine, Faculty of Dentistry, Tabriz University of Medical Sciences, Tabriz, Iran; ^4^ Doctor Irani Clinic and Research Center, Tehran, Iran

**Keywords:** Antibacterial Agents, Enterococcus Faecalis, Eucalyptus, Moringa Oleifera, Plant Extracts

## Abstract

**Background:**

Enterococcus faecalis has been considered as a main factor for rendodontic treatment failure. Herbal products are increasingly used as their antimicrobial efficacy. This study aimed to investigate and compare the in vitro antimicrobial effects of two plants, Moringa oleifera and eucalyptus, with sodium hypochlorite as the standard irrigation solution against Enterococcus faecalis.

**Materials and Methods:**

In the present in-vitro study, thirty mandibular premolars were decoronated, and root canal instrumentation was performed. Each sample was separately autoclaved, inoculated with E. faecalis, and incubated. The samples were divided into six groups based on the type of intervention (n=5 in each group): 1) microemulsion of M. oleifera, 2) microemulsion of eucalyptus, 3) 2.5% sodium hypochlorite, 4) a solution combining both M. oleifera and eucalyptus, 5) positive control, and 6) negative control. The root canals were obturated with gutta-percha and incubated for 90 days. Microbial sampling from the root canals was performed before (S1) and after root canal irrigation (S2), and the viability of microorganisms was reported in terms of the number of colony-forming units.

**Results:**

A significant difference in CFU/mL was observed among all groups (P0.001). The negative control showed complete inhibition, whereas the positive control exhibited 105 CFU/mL. Mean bacterial counts were lowest in the Moringa+Eucalyptus group (4921±3571 CFU/mL), followed by NaOCl (5720 ± 8952 CFU/mL), Eucalyptus (7246±9257 CFU/mL), and Moringa (8053±6247 CFU/mL). Pairwise comparisons revealed no statistically significant differences between the experimental irrigants (P0.05).

**Conclusion:**

M. oleifera and eucalyptus microemulsions exhibited similar antimicrobial effects against E. faecalis, and no synergistic effect of these two extracts was observed.

## Introduction

The goal of root canal treatment is to eliminate microorganisms and their products from the root canal system [1]. Although mechanical instrumentation is one of the most critical steps in root canal treatment, it does not result in the complete removal of microorganisms [2]. Due to the complexity of the canal anatomy, antimicrobial irrigation solutions play a vital role in cleaning and shaping the root canals. These solutions penetrate the dentinal tubules and reach the fine extensions of the root canal. Therefore, antimicrobial irrigants are complementary techniques to mechanical instrumentation [3].

Enterococcus faecalis is a gram-positive anaerobic bacterial species recognized as one of the most resistant infectious species in root canals and is a significant factor in the failure of root canal treatments [4], with a prevalence ranging from 29% to 77% [5][6]. The challenge of eliminating E. faecalis from the root canal system is due to microbial resistance and the microorganism's high capacity to penetrate deep into the dentinal tubules and form biofilms [7]. To reduce the failure rate of treatments, root canal irrigants must also be evaluated for their effectiveness against E. faecalis [8].

Sodium hypochlorite has been introduced as the standard for comparing root canal irrigation solutions, with powerful antimicrobial and tissue-dissolving effects [9][10][11]. However, using this irrigant is limited by some drawbacks, such as cytotoxic effects on periapical tissues, its inability to remove the smear layer, and an unpleasant taste and smell [11][12]. Therefore, the search is underway for an alternative irrigant to sodium hypochlorite with equivalent efficacy but lower toxicity and better patient acceptance.

The complications and the increasing prevalence of antibiotic-resistant strains have prompted researchers to use less toxic and more compatible herbal products for root canal treatment [13]. Studies have shown that extracts from certain plants, such as Moringa oleifera and eucalyptus, can effectively control microbial infections [14][15]. M. oleifera, known as the horseshoe tree, drumstick tree, or miracle tree [16], is indigenous to India; it belongs to the Moringaceae family and has various medicinal properties [17]. Evidence has shown that this plant has antimicrobial, antiviral, anti-sclerotic, and anti-inflammatory effects and is used to treat malnutrition, malaria, colon cancer, and myeloma [18]. This plant's antimicrobial effects are attributed to saponins, flavonoids, tannins, alkaloids, phenols, and triterpenoids, each with mechanisms for controlling microorganisms [19]. Studies have indicated that this plant has favorable antimicrobial effects against E. faecalis [20‒22].

Eucalyptus (Eucalyptus globulus) is recognized as one of the antimicrobial compounds due to its good

biocompatibility and compatibility with endodontic sealers [23][24]. Additionally, due to its effective tissue-dissolving properties, eucalyptus oil has been used as a solvent for sealers [15]. Furthermore, the antimicrobial effect of eucalyptus oil against Streptococcus mutans and E. faecalis has been established [25].

The review of the literature showed that the antimicrobial effects of the oil and extract of these two plants against E. faecalis have been substantiated; however, the antimicrobial effects of their extracts as potential irrigants in root canal treatment have not yet been studied.

## Materials and Methods

### Study Design and Sample Size Determination

This laboratory-based experimental investigation showed its foundation on single-rooted human teeth extracted for orthodontic indications at the Faculty of Dentistry, Tabriz University of Medical Sciences. In addition to orthodontic indications, teeth extracted for periodontal reasons were also included. Only single-rooted human mandibular premolars with fully formed apices and radiographically confirmed single canals were selected. The sample size was calculated using the Power & Sample Size software according to the formula n=(Z1-α/2 + Z1-β)² × (σ1² + σ2²) / (μ1 - μ2)², which indicated suitability for studies comparing two means of a single attribute. Based on data reported by Bankur et al., 2019 (26) regarding inhibition zone diameters for 10% eucalyptus and dimethylformamide against A. actinomycetemcomitans, and considering α=0.05, power=80%, σ1=0.4, σ2=0.98, μ1=1.2, μ2=0.5, with a 10% error margin, 24 specimens were estimated. Because six groups were planned, four samples per group were required, yet five teeth were included in each group to increase reliability. Teeth exhibiting intact crowns, absence of restorations, lack of cracks, and without caries were selected, which indicated proper entry criteria. The teeth were examined visually and under a stereomicroscope (Nikon SMZ1000, Tokyo, Japan) to assure uniformity before inclusion. Samples were stored in 0.9% normal saline until the experiment began.

### Specimen Preparation

Thirty single-rooted teeth meeting the inclusion requirements were used. They were first immersed for 15 min in 5.25% sodium hypochlorite, which showed effective surface debridement, and brushed to remove remaining tissue remnants. After decoronation, using a diamond bur under water cooling to achieve a standardized root length of 14 mm, canal length determination was performed using a size #15 file (Mani, Tochigi, Japan) that was advanced until its tip appeared at the apical foramen, after which 1 mm was subtracted to define the working length. A glide path was created with a #25 file to ensure smooth canal preparation.

Cleaning and shaping were completed using ProTaper rotary files up to F2. During instrumentation, each root canal was irrigated with 4 mL of 2.5% sodium hypochlorite using a disposable syringe. Following preparation, smear layer removal was performed with 3 mL of 5.25% sodium hypochlorite for 3 min, followed by 3 mL of 17% EDTA for 3 min. A final rinse with normal saline removed potential precipitates. The apical foramen was sealed with composite resin before sterilization. Sterilization was performed by autoclaving the roots at 121 °C for 20 min.

Except for the negative control group, teeth were inoculated with Enterococcus faecalis. The strain E. faecalis (ATCC 29212) was cultured on bile esculin agar for 24 h at 37 °C, suspended in BHI broth, and adjusted to 1.5 × 108 CFU/mL (0.5 McFarland). Roots were inoculated with the suspension and incubated for 48 h at 37 °C.

### Preparation of Microemulsions

The Moringa oleifera powder was purchased from orga-life.ir and Eucalyptus globulus powder from giyahkala.com, and both powders were used in the preparation of the microemulsions.

For each microemulsion, 300 mg of the plant powder was mixed with an oil solvent and processed using a sonicator along with Tween 20 and Span 80 surfactants. Sonication ensured thermodynamic stability and resulted in a transparent microemulsion. Additionally, a homogeneous non-aqueous combined solution was prepared by mixing separate plant mixtures and combining them dropwise.

### Grouping and Irrigation Protocols

The teeth were randomly divided into six groups (n=5 per group), which showed even distribution according to intervention type: (1) 2.5% sodium hypochlorite; (2) eucalyptus microemulsion 100% (21) used at 3 mg/mL; (3) Moringa oleifera microemulsion 100% (22) at 3 mg/mL; (4) positive control (bacterial inoculation and saline irrigation); (5) negative control (no bacterial inoculation and saline rinse); and (6) combined eucalyptus-moringa solution. Irrigation was done passively for 30 s with a sterile 27-gauge needle, which indicated similar application across groups. Additionally, 5 mL of each test solution was placed inside the canal for 10 minutes, with the needle positioned 4 mm short of the working length.

All canals were obturated with gutta-percha (Meta Biomed Co., Ltd., Chungbuk, Korea) and AH26 sealer (Dentsply Maillerfer, Ballaigues, Switzerland) using lateral compaction, and specimens were incubated for 90 days at 37 °C to allow microbial penetration testing.

### Microbiological Assessment

After incubation, the apical and coronal thirds were removed, and dentin shavings (2 mg) were collected from the middle third of each root. These samples were transferred into 2 mL of TSB medium (Sigma-Aldrich, St. Louis, MO, USA) in sterile capped tubes and incubated for 48 h at 37 °C. The tubes were then vortexed to ensure homogenization, and 0.1 mL was inoculated onto trypticase soy agar (TSA) plates (Sigma-Aldrich, St. Louis, MO, USA) in three directional streaks from top to bottom, which indicated standardized plating. Plates were incubated for 24 h at 37 °C, and colony-forming units (CFU) were counted using a digital colony counter.

### Statistical Analysis

Data analysis was carried out with IBM SPSS Statistics for Windows, version 21 (IBM Corp., Armonk, N.Y., USA). Owing to non-normal data distribution, the Kruskal-Wallis test was applied for intergroup comparisons, while Mann-Whitney U tests showed pairwise evaluations when needed. A significance level of p ≤ 0.05 indicated meaningful differences.

### Ethical Considerations

The study was initiated after obtaining ethical approval from the Research Ethics Committee of Tabriz University of Medical Sciences. All procedures followed institutional and national guidelines. Because the research used extracted teeth that were removed for clinical reasons, informed consent from donors was not required. The microbiological work was conducted under strict biosafety conditions, which showed adherence to aseptic protocols and ensured a safe environment for all laboratory personnel.

## Results

**Table:** Table1. Comparison of the Antimicrobial Activity of Experimental Irrigants against Enterococcus Faecalis

**Group**	***E. faecalis*** **count (CFU/mL) **	P*
Sodium hypochlorite (NaOCl)	5720 ± 8952	
*Eucalyptus globulus *	7246 ± 9257	
*Moringa oleifera *	8053 ± 6247	<0.01
Moringa + Eucalyptus	4921 ± 3571	
Negative control	0	
Positive control	>100000	

^*^ Kruskal–Wallis test

**Table:** Table2. Pairwise Comparison of Groups based on E. faecalis Counts

**Group I**	**Group J**	**Mean Difference (I−J) **	P*
NaOCl	*Eucalyptus*	−1526	0.349
NaOCl	*Moringa*	−2333	0.156
NaOCl	Moringa+Eucalyptus	799	0.578
*Eucalyptus*	*Moringa*	−807	0.641
*Eucalyptus*	Moringa+Eucalyptus	2325	0.147
*Moringa*	Moringa+Eucalyptus	3132	0.126

^*^ Mann–Whitney U test

This study evaluated and compared the antimicrobial activity of Moringa oleifera extract, Eucalyptus globulus extract, their combination, and sodium hypochlorite (NaOCl) against Enterococcus faecalis inoculated into extracted human teeth. The quantitative assessment of bacterial growth demonstrated clear differences between the experimental and control groups.

### Overall Comparison Between Groups

Based on the Kruskal-Wallis analysis, a statistically significant difference was observed among the six study groups with respect to E. faecalis colony-forming units (CFU/mL) (P<0.001). As summarized in Table-1, the negative control group exhibited complete inhibition of bacterial growth, confirming the sterility of the samples and the reliability of experimental procedures. In contrast, the positive control group demonstrated extremely high bacterial counts (>10^5 CFU/mL), verifying the viability of the microorganism and the appropriateness of the contamination model.

Among the four test groups, the lowest mean bacterial count was observed in the combination group (Moringa + Eucalyptus) (4921 ± 3571 CFU/mL), followed by NaOCl (5720 ± 8952 CFU/mL). The Eucalyptus extract (7246 ± 9257 CFU/mL) and Moringa oleifera extract (8053 ± 6247 CFU/mL) showed slightly higher bacterial counts; however, these values remained markedly lower than those of the positive control.

### Pairwise Comparisons

To determine whether any of the irrigants differed significantly from one another, pairwise comparisons were conducted using the Mann-Whitney U test. The results (Table-2) indicate that none of the differences in mean CFU/mL between any two experimental groups reached statistical significance (P>0.05 for all comparisons). These findings demonstrate that Moringa oleifera, Eucalyptus globulus, their combined extract, and NaOCl exhibited comparable antimicrobial properties against E. faecalis under the conditions of this study.

Although NaOCl and the Moringa + Eucalyptus combination numerically showed lower bacterial counts, these reductions were not significantly different from those produced by either Moringa or Eucalyptus alone. This suggests that all four solutions are similarly effective.

## Discussion

Our in vitro investigation aimed to comparatively evaluate the antimicrobial efficacy of Moringa oleifera and eucalyptus microemulsions against this resilient bacterium, positioning them against the established standard of care, sodium hypochlorite (NaOCl). Our results demonstrated a substantial reduction in E. faecalis viability across all tested irrigant groups compared to the positive control, with our experimental herbal extracts showing comparable efficacy to NaOCl and exhibiting no statistically significant difference between them. Systematic review study by Ruksakiet et al. found significant antimicrobial efficacy of NaOCl in root canal disinfection [27], that shows the roubustness of our study results when it showed similar effects of evaluated herbs compared to NaOCl. But on the other hand, Estrela et al. conducted a systematic review and concluded that NaOCl exhibit low ability to eliminate E. faecalis when evaluated using PCR or culture techniques that indicates inherent difficulty in completely eradicating this organism from root canal systems using conventional methods [28]. This shows that such single study results may need further exploration for clinical usage.

In another report, Panchal et al. [29] reported that 1.25% eucalyptus extract outperformed 5.25% NaOCl in reducing E. faecalis counts in root canals that was suggesting that concentration and formulation (microemulsion vs. simple extract) might significantly influence observed efficacy. Also studies by Raoof et al. [30][31] which, while confirming the antibacterial effect of eucalyptus extracts (Eucalyptus galbie), found no significant enhancement in activity when combined with calcium hydroxide powder over the positive control or even compared directly to NaOCl in some cases, as also evident in Nourzadeh et al. study [32]. This discrepancy may reflect differences in the specific plant species, extract concentration, testing methodology, or the nature of the combination tested. Our study similarly found no synergistic effect when the two extracts were combined that is showing the idea that complex mixtures may not necessarily offer additive or synergistic advantages for this particular challenge organism. This observation is further supported by Mathew et al. [33], who developed an indigenously prepared herbal extract (EndoPam, containing eucalyptus) and found it comparable to NaOCl in ex vivo studies; it was suggesting potent individual herbal components can suffice without necessarily needing combination therapies for effectiveness against E. faecalis.

Our findings are consistent with previous research evaluating Moringa oleifera extracts. For example, Alharbi et al. [34] reported that Moringa oleifera leaf extract exhibited antibacterial efficacy against E. faecalis similar to OCT and NaOCl in an in vitro setting. Furthermore, studies focusing specifically on Moringa oleifera, such as Natsir et al. [35] and Shafiq et al. [36], have showed its benefits for endodontic use, including smear layer removal efficacy and rich phytochemical composition (high in flavonoids and phenolics) known to possess antimicrobial properties, thereby supporting the observed antibacterial activity. While our study focused solely on the immediate post-irrigation antibacterial effect, the sustained reduction in bacterial load over the 90-day incubation period observed in the herbal groups warrants further investigation for their long-term stability and residual efficacy within the complex root canal environment. Also, Sopandani et al. [21], who demonstrated that 75% and 100% extracts of Moringa oleifera plant were effective in eliminating E. faecalis. Furthermore, Hijar et al. [37] showed that the methanolic extract of M. oleifera had a significant antimicrobial effect against E. faecalis within 48 hours, with no toxicity against epithelial cell lines.

This study had many limitations. First, it was conducted on extracted single-rooted teeth with straight roots, and the different morphologies and anatomies of root canals were not examined. The study did not investigate microorganisms in other anatomical areas, such as dentinal tubules. Additionally, this laboratory study did not assess the impact of physiological factors on the studied formulations. Future studies are also recommended to focus more on chelating agents and acidifiers in combination with the mentioned formulations.

## Conclusion

Despite the mentioned limitations, it can be concluded that the 100% microemulsion formulations of both M. oleifera and eucalyptus, either alone or in combination, exhibited a significant antimicrobial effect against E. faecalis in extracted teeth, comparable to the antimicrobial effect of a 2.5% sodium hypochlorite solution. Therefore, it can be concluded that plant formulations, both alone and in combination, exhibit beneficial antimicrobial effects compared to intracanal irrigation solutions.

## Conflict of Interest

None.
